# Proteinuria as a Biomarker for COVID-19 Severity

**DOI:** 10.3389/fphys.2021.611772

**Published:** 2021-03-09

**Authors:** Hajar Ouahmi, Johan Courjon, Lucas Morand, Juliette François, Vincent Bruckert, Romain Lombardi, Vincent Esnault, Barbara Seitz-Polski, Elisa Demonchy, Jean Dellamonica, Sonia Boyer-Suavet

**Affiliations:** ^1^Service de Médecine Intensive Réanimation, CHU de Nice, Université Côte d’Azur, Nice, France; ^2^Service de Néphrologie-Dialyse-Transplantation, CHU de Nice, Université Côte d’Azur, Nice, France; ^3^Service d’Infectiologie, CHU de Nice, Université Côte d’Azur, Nice, France; ^4^Université Côte d’Azur, Centre Méditerranéen de Médecine Moléculaire (C3M), CHU de Nice, INSERM U1065, Nice, France; ^5^Service de Réanimation CH Antibes-Juan les Pins, Antibes, France; ^6^Département de réanimation médico-chirugicale et transplantation d’organe, hôpital L’Archet 2, CHU de Nice, Université Côte d’Azur, Nice, France; ^7^Laboratoire d’Immunologie, CHU de Nice, Université Côte d’Azur, Nice, France; ^8^Centre de référence Maladies rares Syndrome néphrotique idiopathique, CHU de Nice, Université Côte d’Azur, Nice, France; ^9^Unité de Recherche Clinique de la Côte d’Azur (UR2CA), Université Côte d’Azur, Nice, France

**Keywords:** acute kidney injury, biomarker, COVID-19, proteinuria, SARS-CoV-2, kidney involvement, pronostic and predictive factors

## Abstract

**Background:**

Renal involvement in syndrome coronavirus 2 (SARS-CoV-2) infection has been retrospectively described, especially acute kidney injury (AKI). However, quantitative proteinuria assessment and its implication in coronavirus disease 2019 (COVID-19) remain unknown.

**Methods:**

In this prospective, multicenter study in France, we collected clinical and biological data including urinary protein to creatine ratio (UPCR) in patients presenting with moderate to severe COVID-19. Clinical outcome was analyzed according to the level of UPCR.

**Results:**

42/45 patients (93.3%) had renal involvement (abnormal urinary sediment and/or AKI). Significant proteinuria occurred in 60% of patients. Urine protein electrophoresis showed tubular protein excretion in 83.8% of patients with proteinuria. Inflammatory parametersand D-dimer concentrations correlated with proteinuria level. Patients who required intensive care unit (ICU) admission had higher proteinuria (*p* = 0.008). On multivariate analysis, proteinuria greater than 0.3 g/g was related to a higher prevalence of ICU admission [OR = 4.72, IC95 (1.16–23.21), *p* = 0.03], acute respiratory distress syndrome (ARDS) [OR = 6.89, IC95 (1.41–53.01, *p* = 0.02)], nosocomial infections [OR = 3.75, IC95 (1.11–13.55), *p* = 0.03], longer inpatient hospital stay (*p* = 0.003).

**Conclusion:**

Renal involvement is common in moderate to severe SARS-CoV-2 infection. Proteinuria at baseline is an independent risk factor for increased hospitalization duration and ICU admission in patients with COVID-19.

## Introduction

Coronavirus disease 2019 (COVID-19) is a transmitted disease caused by the novel severe acute respiratory syndrome coronavirus 2 (SARS-CoV-2). It primarily manifests itself as an acute respiratory illness but can affect multiple organs such as kidneys, heart, digestive tract, and nervous system (Wang et al., 2020). In previous reports of SARS and Middle East Respiratory Syndrome coronavirus, acute kidney injury (AKI) was described in 5 – 15% of patients and was associated with high mortality rates (60–90%) (Chu et al., 2005; Cha et al., 2015). Recent reports showed renal abnormalities in COVID-19 patients (Naicker et al., 2020), but kidney involvement has not been yet well characterized (only retrospective cohorts using urine dip-stick tests) (Pei et al., 2020; Wang et al., 2020). A recent Chinese study also reported that AKI was an independent risk factor for mortality (Cheng et al., 2020). However, the exact mechanism of kidney involvement remains unclear: sepsis-related cytokine storm (Mehta et al., 2020) or direct cellular injury induced by the virus (Sun et al., 2020). Human angiotensin-converting enzyme 2 (ACE2) receptor has been identified as the functional receptor for SARS-CoV-2 and is highly expressed in kidneys (Hoffmann et al., 2020; Walls et al., 2020). These data suggest that the kidney might be a target of this SARS-CoV-2 as highlighted in pathological examinations (Su et al., 2020). Reports about the natural course of renal complications during SARS-CoV-2 are scarce, most of them concern only AKI or specific populations (e.g., Chinese) (Cheng et al., 2020; Pei et al., 2020), or kidney transplant recipients (Akalin et al., 2020; Alberici et al., 2020; Banerjee et al., 2020). We aimed to prospectively identify renal involvement, more especially proteinuria (quantitative) at baseline and its prognosis in a French cohort with moderate to severe SARS-CoV-2 infection.

## Materials and Methods

### Study Protocol

Multicenter prospective observational study.

### Study Approval

Use of routinely collected health data as a current care practice study (NCT04355624).

### Patient Population

Inclusion criteria: all patients aged ≥18 years-old with symptomatic proven moderate to severe COVID-19 according to the WHO classification, admitted in one infectious diseases department or in three different intensive care unit (ICU) of Nice University Hospital and Antibes Juan-les-Pins hospital general hospital, between March 15th to April 19th, 2020.

Patients with a history chronic kidney disease and pregnant women were non-included.

Patients with documented urinary tract infection at inclusion were excluded to avoid confusing results.

### Data Sources

The demographic characteristics (medical history, clinical symptoms, laboratory data, and medications) were extracted from electronic medical records. Urinary data were collected on urine sample: microalbuminuria, urine protein/creatinine ratio (UPCR), urine protein electrophoresis, red blood, and white blood cells counts.

### Definitions

Proven COVID-19 was defined as a positive SARS-CoV-2 real-time polymerase chain reaction (RT-PCR) assay in nasopharyngeal swabs.

Severe COVID-19 was defined as respiratory failure or need for mechanical ventilation, shock or organ failure or need for ICU admission.

Acute kidney injury was defined according to KDIGO guidelines.

The date of disease onset was defined as the day of first symptom.

The last day of follow up was the in-hospital death or hospital discharge.

### Statistical Analysis

For descriptive statistics, data are presented as median (ranges) or mean ± standard deviation. Shapiro-Wilk test was used to test for normal distribution of variable. Comparison of qualitative criteria was performed using Chi-square test or Fisher’s exact test. Comparison of quantitative variables was performed using the Student *t*-test or Wilcoxon-Mann–Whitney test. Spearman rank-order test was used to find correlations between continuous variables. Multivariate analysis was performed using logistic regression. The multivariate model was built by including variables that met the 20% significance threshold in univariate analysis. Choice among colinear variables was performed using Akaike Iteration Criteria. Receiver Operating Characteristic (ROC) curve was used to evaluate the performance of the test. Comparisons for survival curves were performed using Kaplan-Meier analysis. A *p*-value < 0.05 indicated statistical significance. Statistical analysis was performed using GraphPad Prism 5.0 (GraphPad Software, Inc., San Diego, CA, United States) and RStudio [RStudio Team (2019). RStudio. Inc., Boston].

## Results

### Baseline Characteristics

A total of 45 patients were enrolled and followed for a median of 11 days (5–23 days). Twenty-two patients (49%) developed severe COVID-19 and required ICU admission. Table 1 shows the characteristics of the cohort. Prevalence of hypertension and diabetes mellitus was 37.8 and 26.7%, respectively. Less than 25% of patients were on renin-angiotensin system (RAS) blockers. Two patients died from SARS-CoV-2 infection: one in the ICU from refractory respiratory failure, one patient died from respiratory failure in conventional unit with DNR order.

**TABLE 1:** Clinical and biological characteristics of the cohort.

Variables	All patients (*n* = 45)	Severe COVID-19 (*n* = 22)	Non-severe COVID-19 (*n* = 23)	*P-*value
**Clinical characteristics**				
Age, years	64.0 (48.5–71.5)	66.0 (58.8–72.0)	58 (47.0–68.0)	0.11
Male patients, *n* (%)	31.0 (68.9)	18.0 (81.8)	13.0 (56.5)	0.07
Respiratory disease, *n* (%)	9.0 (20.0)	5.0 (22.7)	4.0 (17.4)	0.65
Hypertension, *n* (%)	17.0 (37.8)	9.0 (40.9)	8.0 (34.8)	0.67
Diabetes mellitus, *n* (%)	12.0 (26.7)	6.0 (27.3)	6.0 (26.1)	0.93
Cardiac disease, *n* (%)	5.0 (11.1)	3.0 (13.6)	2.0 (8.7)	0.60
Vascular disease, *n* (%)	3.0 (6.7)	1.0 (45.5)	2.0 (8.7)	0.58
Active and former smokers, *n* (%)	13.0 (28.9)	9.0 (39.1)	4.0 (17.4)	0.08
Immunosuppression, *n* (%)	6.0 (13.3)	3.0 (13.6)	3.0 (13)	0.95
ACE inhibitor, *n* (%)	1.0 (22.2)	1.0 (4.5)	0.0	0.30
ARB, *n* (%)	8.0 (17.8)	2.0 (9.0)	6.0 (26.1)	0.14
BMI (kg/m^2^)	27.8 (24.3–32.5)	28.9 (25.0–32.9)	25.8 (23.4–32.6)	0.47
Systolic blood pressure, mmHg	125.0 (120.0–137.5)	128.5 (120.0–144.0)	120.0 (115.0–130.0)	0.03*
Diastolic blood pressure, mmHg	75.0 (67.0–80.0)	80.0 (71.5–80.0)	70.0 (60.0–80.0)	0.02*
Days from illness onset to admission	8.0 (5.0–12.0)	11.0 (6.0–14.0)	7.0 (4.0–10.0)	0.03*
**Biological data**				
Leukocytes, G/L	5.7 (4.4–8.3)	7.7 (5–9.3)	4.9 (4–6.9)	0.03*
Lymphocytes, G/L	0.9 (0.6–1.2)	0.7 (0.5–1)	1 (0.7–1.3)	0.11
Neutrophils, G/L	4.7 (3.2–6.8)	5.9 (4.2–7.9)	3.4 (2.5–4.9)	0.008*
Hemoglobin, G/L	13.0 (12.2–14.2)	13.0 (12.1–14.2)	13.2 (12.2–14.2)	0.93
Platelets, G/L	205.0 (165.5–270.0)	255.5 (174.8–288.5)	203.0 (157.0–256.0)	0.11
D-dimer, ng/ml	1764.0 (779.0–5689.0)	4944.0 (1814.0–9374.0)	913.0 (476.0–1422.0)	<0.001*
Fibrinogen, g/l	7.1 (5.8–8.7)	7.8 (6.8–9.7)	5.5 (4.8–7.4)	0.001*
Procalcitonin, ng/ml	0.2 (0.1–0.5)	0.4 (0.2–0.8)	0.1 (0.1–0.3)	0.002*
C-reactive protein, mg/l	87.8 (57.3–150.9)	123.3 (80.3–219.2)	83.6 (41.1–112.8)	0.02*
Lactose deshydrogenase, U/L	666.0 (488.0–790.5)	677.0 (490.0–992.3)	649.0 (470.0–694.0)	0.15
Sodium, mmol/L	137.0 (134.0–139.0)	135.5 (133.0–141.0)	138.0 (135.0–139.0)	0.24
Potassium, mmol/l	4.0 (3.7–4.3)	4.2 (3.8–4.5)	3.8 (3.6–4.1)	0.04*
Bicarbonate, mmol/l	23.0 (22.0–25.0)	22.0 (20.0–23.3)	24.0 (23.0–26.0)	0.003*
Albumin, g/l	28.9 (25.4–32.8)	27.0 (24.7–30.0)	30.8 (27.3–34.3)	0.02*
BUN, mmol/l	5.2 (3.9–7.3)	4.7 (2.8–6.3)	3.7 (1.7–4.6)	0.06
Creatinine, μmol/l	72.0 (57.0–87.5)	75.5 (59.3–94.0)	72.0 (55.0–82.0)	0.44
eGFR, ml/min per 1.73 m^2^	87 (82–101)	89 (80–97)	87 (82–106)	0.45
**Renal involvement**				
AKI, *n* (%)	12.0 (26.7)	8.0 (36.4)	4.0 (17.4)	0.15
KDIGO 1	6/12 (50.0)	4/8 (50)	2/4 (50)	
KDIGO 2	4/12 (33.3)	2/8 (25)	2/4 (50)	
KDIGO 3	2/12 (16.7)	2/8 (25)	0	
RRT	2/12 (16.7)	2/8 (25)	0	
Proteinuria, g/g	0.50 ± 0.47	0.59 ± 0.40	0.42 ± 0.53	0.008*
Proteinuria >0.3 g/g, *n* (%)	27 (60.0)	17 (77.3)	10 (43.5)	0.02*
Non-albumin proteinuria, *n* (%)	37 (82.2)	18 (81.8)	19 (82.6)	0.94
Microalbuminuria, *n* (%)	5 (11.1)	3 (13.6)	2 (8.7)	0.6
Hematuria, *n* (%)	21 (46.7)	12 (54.5)	9 (39.1)	0.3
Leukocyturia, *n* (%)	21 (46.7)	13 (59.1)	8 (34.8)	0.10
Normoglycemic glycosuria, *n* (%)	0	0	0	
**Treatment**				
Steroids, *n* (%)	22 (48.9)	17 (77.3)	5 (21.7)	<0.001*
Antibiotics, *n* (%)	21 (46.7)	17 (77.3)	4 (17.4)	0.001*
Lopinavir/Ritonavir, *n* (%)	3 (6.7)	3 (13.6)	0	0.07
Hydroxychloroquine, *n* (%)	17 (37.8)	14 (63.6)	3 (13.0)	<0.001*
Tocilizumab, *n* (%)	1 (2.2)	1 (4.5)	0	0.97
Vasopressor, *n* (%)	12 (26.7)	12 (54.5)	12 (52.2)	0.001*
Invasive ventilation, *n* (%)	15 (33.3)	15 (68.1)	0	0.001*
**Outcome**				
Nosocomial infection, *n* (%)	15 (33.3)	13 (59.1)	2 (8.7)	<0.001*
In-hospital death, *n* (%)	2 (4.4)	1 (4.5)	1 (4.3)	0.97
Length of stay, days	11 (5–23)	23 (14–32)	7 (5–11)	0.001*

ACE, angiotensin-converting-enzyme inhibitor; ARB, angiotensin II receptor blockers; BMI, body mass index; BUN, blood urea nitrogen; AKI, acute kidney injury; RRT, renal replacement therapy. *Significant, P < 0.05.

### Renal Involvement

On admission, 93.3% of patients (42 of 45) presented with renal involvement.

### Urine Sediment Abnormalities

Significant proteinuria (>0.3 g/g) occurred in 27 patients (60%) with a mean of 0.50 ± 0.47 g/g (77.3% patients in the severe disease group versus 43% in the non-severe group, *p* = 0.02). Higher UPCR was observed in patients with severe COVID-19 (0.59 ± 0.40 versus 0.42 ± 0.53 g/g, *p* = 0.008). Urine protein electrophoresis showed non-albumin proteinuria excretion in 77.7% of patients with significant proteinuria. Inflammatory variables, D-dimers and length of hospital stay were correlated with the level of proteinuria (Supplementary Table 1).

### Acute Kidney Injury

The incidence of AKI in the overall cohort was 26.7% (12 of 45 patients) according to KDIGO definition (Table 1). Stage 1 AKI accounted for 50% (6/12 patients with AKI), stage 2 comprised 33.3% (4/12), and 16.7% (2/12) reached stage 3 and required renal replacement therapy (RRT). Among patients with AKI: proteinuria, hematuria and leukocyturia were not different compared with non-AKI patients (Supplementary Table 2). Mortality was not different between AKI and non-AKI patients. Among patients in whom AKI developed, 50% recovered.

### Impact of Proteinuria in SARS-CoV-2 Infection

Patients who required ICU admission had significantly higher level of proteinuria (0.59 ± 0.40 versus 0.42 ± 0.53 g/g, *p* = 0.008).

**FIGURE 1:**
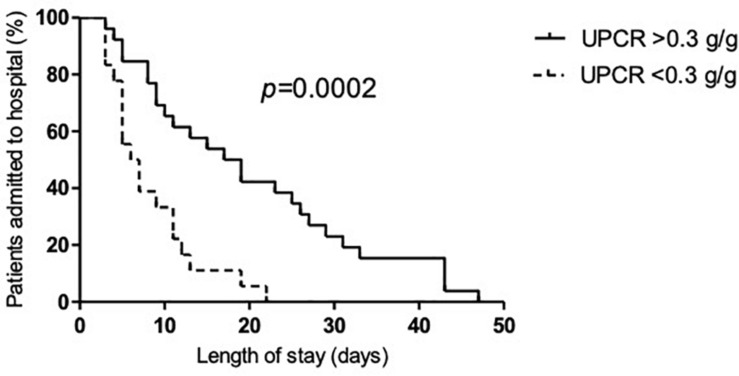
Significant proteinuria is associated with an increased risk to develop severe disease and longer hospitalization in patients with COVID-19 disease. Patients with higher proteinuria presented an increased risk to develop severe disease and to require admission to the intensive care unit. Kaplan-Meier analysis of the effect of proteinuria >0.3 g/g on length of stay in patients hospitalized with SARS-CoV-2 infection showed that patients with proteinuria greater than 0.3 g/g presented a longer hospitalization (log-rank *p* = 0.0002).

Patients with significant proteinuria defined as proteinuria above 0.3 g/g had a longer hospital stay [19 days (9–31) versus 7 days (5–11), *p* = 0.001], a higher prevalence of nosocomial infection (48.1 versus 11.1%, *p* = 0.01) and acute respiratory distress syndrome (ARDS) (48.1 versus 11.1%, *p* = 0.01) but mortality was not significantly higher (*p* = 0.24) (Table 2). Using univariate and multivariate analyses, proteinuria was related to a higher prevalence of ICU admission, ARDS and nosocomial infection (Table 3). Kaplan-Meier analysis revealed a significantly longer hospitalization duration for patients with a significant proteinuria (Figure 1). For in-hospital mortality and AKI, proteinuria was not an independent predictor (Table 3).

**TABLE 2:** Characteristics of patients with and without proteinuria >0.3 g/g.

Variable	Proteinuria (*n* = 27)	No proteinuria (*n* = 18)	*P*-value
Age, years	66.0 (49.0–72.0)	61.0 (43.5–68.3)	0.18
Male patients, *n* (%)	22 (81.5)	9 (50.0)	0.03*
Day from illness onset to admission, days	9.0 (5.0–11.0)	8.0 (5.5–12.5)	0.83
Systolic blood pressure, mmgh	127.0 (120.0–140.0)	120.0 (120.0–130.0)	0.40
Diastolic blood pressure, mmgh	80.0 (70.0–80.0)	70.0 (60.0–80.0)	0.54
Respiratory disease, *n* (%)	7 (25.9)	2 (11.1)	0.22
Hypertension, *n* (%)	11 (40.7)	6 (33.3)	0.62
Diabetes mellitus, *n* (%)	10 (37.0)	2 (11.1)	0.05
Cardiac disease, *n* (%)	3 (11.1)	2 (11.1)	1
Vascular disease, *n* (%)	3 (11.1)	0	0.14
Active and former smokers, *n* (%)	9 (33.3)	4 (22.2)	0.42
ACEI, *n* (%)	1 (3.7)	0	0.41
ARB, *n* (%)	3 (0.11)	5 (27.8)	0.15
BMI (Kg/m^2^)	26.5 (24.3–32.9)	28.3 (24.1–32.6)	0.74
C Reactive protein, mg/l	112.8 (75.1–168.1)	51.6 (81.2–104.0)	0.04*
Procaciltonin, ng/ml	0.3 (0.1–0.7)	0.2 (0.1–0.4)	0.27
D–Dimer ng/ml	4349.0 (1701.0–6828.0)	779.0 (472.0–1501.0)	<0.001*
AKI, *n* (%)	9 (33.3)	3 (16.7)	0.22
ICU admission, *n* (%)	17 (62.9)	5 (27.8)	0.02*
ARDS, *n* (%)	13 (48.1)	2 (11.1)	0.01*
Length of stay, days	19 (9.0–31.0)	6.5 (4.8–11.3)	0.001*
Nosocomial infection, *n* (%)	13 (48.1)	2 (11.1)	0.01*
In-hospital death, *n* (%)	2 (7.4)	0	0.24

ACE, angiotensin-converting-enzyme inhibitor; ARB, angiotensin II receptor blockers; ARDS, acute respiratory distress syndrom; BMI, body mass index; AKI, acute kidney injury; ICU, intensive care unit. *Significant, P < 0.05.

**TABLE 3:** Association between significant proteinuria and outcomes.

Variable	Univariate analysis			Multivariate analysis		
		
	Odds ratio	95% Confidence interval	*P* value	Odds ratio	95% Confidence interval	*P* value
AKI	2.33	0.57–12.02	0.26			
RRT	5.33	0.48–119.84	0.18			
ARDS	6.96	1.55–49.87	0.02*	6.89	1.41–53.01	0.02*
ICU admission	4.08	1.15–16.19	0.03*	4.72	1.16–23.21	0.03*
Vasopressor	11.0	1.82–213.24	0.03*	12.32	1.83–254.97	0.03*
Nosocomial infection	6.96	1.56–49.87	0.02*	3.75	1.11–13.55	0.03*
In-hospital death	4.17	0.63–34.32	0.13			

AKI, acute kidney injury; RRT, renal replacement therapy; ARDS, acute respiratory distress syndrome; ICU, intensive care unit. *Significant, P < 0.05.

## Discussion

Among the 45 patients prospectively analyzed in the present study, a high proportion presented renal involvement. Proteinuria at baseline was associated with poor outcome in SARS-CoV-2 infection. Our data are consistent with a recent retrospective study among 333 patients in China (Pei et al., 2020). Our study confirmed that kidney involvement is common in hospitalized COVID-19 patients, but not only in severe cases. AKI has been previously described as associated with in-hospital mortality in COVID-19 (Cheng et al., 2020) and renal complications in COVID-19 remains associated with poor outcome (Pei et al., 2020). To our knowledge, this is the first study using quantitative assessment of urinary protein excretion and identifying proteinuria as a factor associated with poor outcome in COVID-19 (higher prevalence of ARDS, nosocomial infection, ICU admission). Furthermore, we could show that a significant proteinuria (i.e., above 0.3 g/g) was associated with a higher risk of ICU admission, developing ARDS, requiring vasopressor, developing nosocomial infections. Proteinuria higher than 0.3 g/g was a predictor for length of stay. Similar results have been described in a retrospective cohort with bacterial community-acquired pneumonia (Spoorenberg et al., 2012) but also in non-infectious diseases such as cirrhosis (Lin et al., 2014) or lung cancer (Hsu et al., 2018).

The tubular profile of kidney involvement we describe is consistent with recent pathologic reports (Su et al., 2020). The analysis of 26 autopsies from COVID-19 patients showed diffuse proximal tubule injury with the loss of brush border, non-isometric vacuolar degeneration, necrosis; hyaline casts and microthrombi were also observed. Interestingly, in this study, some patients did not present AKI according to KDIGO definition suggesting subclinical kidney injury. Proteinuria appears as a sensitive tool, comparing to creatinine and/or BUN, for renal assessment in COVID-19, even in subclinical kidney injury.

In our study, hematuria and leukocyturia were present in almost half of the patients but we found no difference between severe and non-severe COVID-19. Our results suggest an interstitial participation in kidney. Histopathologic reports did not show interstitial inflammation but 61.5% of patients had steroids before kidney biopsy was performed (and data are not available for 23% of patients) (Su et al., 2020). Even if a case of collapsing glomerulopathy has been reported in COVID-19 (Gaillard et al., 2020), acute tubular necrosis (ATN) with interstitial component probably remains the main cause of kidney injury. Other factors may contribute to kidney involvement: systemic inflammation and cytokine storm (Burton and Harris, 1996; Coletta et al., 2000; Gohda et al., 2001; D’Amico and Bazzi, 2003; Imai, 2003; Thorevska et al., 2003; Mäkelä et al., 2004; Wassmann et al., 2004; Kielar et al., 2005; Abbate et al., 2006; Liu et al., 2007; Delvaeye and Conway, 2009; Murugan et al., 2010; Ranganathan et al., 2013; Darmon et al., 2014, 2017; Su et al., 2017; Sarhan et al., 2018; Meissner et al., 2019; Cremoni et al., 2020; Mehta et al., 2020; Pan et al., 2020; Rossi et al., 2020; Siddiqi and Mehra, 2020; Tang et al., 2020; Vaninov, 2020; Zhang et al., 2020; Ruetsch et al., 2021), release of pathogen-associated molecular patterns (PAMPs), high levels of damage-associated molecular proteins (DAMPS) from lung injury and severe hypoxemic respiratory failure (Burton and Harris, 1996; Coletta et al., 2000; Gohda et al., 2001; D’Amico and Bazzi, 2003; Thorevska et al., 2003; Mäkelä et al., 2004; Wassmann et al., 2004; Kielar et al., 2005; Abbate et al., 2006; Delvaeye and Conway, 2009; Ranganathan et al., 2013; Darmon et al., 2014, 2017; Su et al., 2017; Sarhan et al., 2018; Meissner et al., 2019; Cremoni et al., 2020; Pan et al., 2020; Rossi et al., 2020; Siddiqi and Mehra, 2020; Tang et al., 2020; Vaninov, 2020; Zhang et al., 2020; Ruetsch et al., 2021). All these factors could also lead to tissue factor release and the hypercoagulate state described in SARS-COV-2 infection (Delvaeye and Conway, 2009; Tang et al., 2020). In our cohort, D-dimer correlated with higher proteinuria and severe disease. Moreover, reports suggested that patients with COVID-19 presented an increased risk for thrombosis or microangiopathy in autopsy reports (Tang et al., 2020). This hypercoagubility might participate in kidney involvement in COVID-19.

Proximal tubules injury has been described (Su et al., 2020), but our results don’t support Fanconi’s syndrome. Microalbuminuria was present in 11.1% of the cohort. High prevalence of microalbuminuria has been described in critically ill patients and is associated with mortality (Thorevska et al., 2003) but here, no difference was found in microalbuminuria according to disease severity. This is the reason why we focused on significant proteinuria. Immunostaining with SARS-CoV-2 nucleoprotein antibody was found positive in tubules and podocytes (Su et al., 2020). One mechanism of kidney impairment may be direct viral infection of renal epithelium, but not all biopsy specimens found viral genetic material and cytopathic effects (Rossi et al., 2020). Mechanisms for proteinuria may result from a defect in proximal tubular resorption and also from impairment of glomerular permeability due to pathophysiologic changes due to pro-inflammatory cytokines (Chu et al., 2005). Proteinuria in COVID 19 could not be differentiate from febrile proteinuria. As in septic state, proteinuria could be predictive of ICU survival. We found higher C-reactive protein levels with higher proteinuria, SARS-CoV-2 infection is known to induce cytokines storm and this pro-inflammatory disorder has been associated with severity and poor outcome (Mehta et al., 2020; Vaninov, 2020; Zhang et al., 2020). Pro-inflammatory cytokines might play a role as previously explored in AKI (Liu et al., 2007; Murugan et al., 2010) but not in proteinuria. Interestingly, the entire cohort was studied during the hyperinflammatory phase of the disease (Siddiqi and Mehra, 2020). These facts can support the relation with proteinuria and cytokine storm in SARS-CoV-2 infection. As described previously, inflammatory cytokines IL1β, IL6, IL8, and TNFα increased in the plasma of moderate and severe COVID-19 patients (Ruetsch et al., 2021). Cytokine storm may contribute to COVID-19 AKI by cooperating with renal resident cells and promoting tubular and endothelial dysfunction. Previous studies described a relevant role for IL6 (Ruetsch et al., 2021). Interleukin-6 can lead renal endothelial cells to produce pro-inflammatory cytokines and chemokines, and can induce kidney vascular permeability, acting in microcirculatory dysfunction. Pro-inflammatory cytokines can also induce capillary leak syndrome and the production of thrombosis. Interestingly, IL6 could be produced by renal resident cells, including podocytes (Burton and Harris, 1996; Coletta et al., 2000; Gohda et al., 2001; Mäkelä et al., 2004; Wassmann et al., 2004; Kielar et al., 2005; Abbate et al., 2006; Ranganathan et al., 2013; Su et al., 2017; Cremoni et al., 2020; Pan et al., 2020; Ruetsch et al., 2021), mesangial cells (Coletta et al., 2000; Gohda et al., 2001), endothelial cells (Wassmann et al., 2004) and tubular epithelial cells (Kielar et al., 2005; Ranganathan et al., 2013). All these cells would actively respond to IL6 (Su et al., 2017). Moreover, plasma and urinary IL6 concentrations correlated with proteinuria in acute Hantavirus-induced nephritis (Mäkelä et al., 2004) and increased IL6 plasma levels and a TH17 profile have already been described in glomerular diseases such as membranous nephropathy (Cremoni et al., 2020).

This study has several limitations. One is the number of patients studied, nevertheless all consecutive patients were prospectively screened in different wards and hospitals strengthening the results obtained. This small sample size could explain our non-significative results on correlating proteinuria to AKI and mortality. Secondly, we could not detect SARS-CoV-2 in urine samples but this is not performed routinely and kidney involvement in SARS-CoV-2 infection seems multifactorial and not only due to viral infection. Last, we did not have renal histopathological data to correlate to biological data since there were no formal indication for kidney biopsy.

Nevertheless, our study is prospective and multicentric. This is the first conducted in an European population. Differences have been described between Caucasian and Asian populations in kidney disease and also in ACE2 expression (Hoffmann et al., 2020). We used quantitative urinary protein excretion that is more accurate but still an easy and inexpensive test in contrast with other urinary markers.

In conclusion, kidney involvement in SARS-CoV-2 infection is common and not only in severe forms. Renal impairment in SARS-CoV-2 infection and more precisely proteinuria is an independent predictor for length of stay and admission to the ICU. Proteinuria is an easily measurable marker to predict outcome and may be used to assess the severity of SARS-CoV-2 infection. Evidence suggest that proteinuria is a marker of chronic disease progression (Burton and Harris, 1996; Abbate et al., 2006). In SARS-CoV-2 infection, quantitative proteinuria should be monitored at admission and during follow-up, even in patients without AKI or severe disease to assess long-term implication of SARS-CoV-2 infection.

## Data Availability Statement

The original contributions presented in the study are included in the article/Supplementary Material, further inquiries can be directed to the corresponding author/s.

## Ethics Statement

Ethical review and approval was not required for the study on human participants in accordance with the local legislation and institutional requirements. The patients/participants provided their written informed consent to participate in this study.

## Collaborators

Mathieu Buscot: Service de Médecine Intensive Réanimation, CHU de Nice, Université Côte d’Azur, Nice, France; Clément Saccheri: Service de Médecine Intensive Réanimation, CHU de Nice, Université Côte d’Azur, Nice, France; Hervé Hyvernat: Service de Médecine Intensive Réanimation, CHU de Nice, Université Côte d’Azur, Nice, France; Denis Doyen: Service de Médecine Intensive Réanimation, CHU de Nice, Université Côte d’Azur, Nice, France; Nihal Martis: Service de Médecine Intensive Réanimation, CHU de Nice, Université Côte d’Azur, Nice, France; Yanis Kouchit: Service de Médecine Intensive Réanimation, CHU de Nice, Université Côte d’Azur, Nice, France; Thomas Citti: Service de Médecine Intensive Réanimation, CHU de Nice, Université Côte d’Azur, Nice, France; Éric Cua: Service d’Infectiologie, CHU de Nice, Université Côte d’Azur, Nice, France; Véronique Mondain: Service d’Infectiologie, CHU de Nice, Université Côte d’Azur, Nice, France; David Chirio: Service d’Infectiologie, CHU de Nice, Université Côte d’Azur, Nice, France; Karine Risso: Service d’Infectiologie, CHU de Nice, Université Côte d’Azur, Nice, France; Philippe Deswardt: Service de Réanimation CH Antibes-Juan les Pins, Antibes, France; Cécilia Benard: Service de Réanimation CH Antibes-Juan les Pins, Antibes, France; Marine Clavaud: Département de réanimation médico-chirugicale et anesthésie, CHU de Nice, Université Côte d’Azur, Nice, France; Abdlazize Sahraoui: Département de réanimation médico-chirugicale et anesthésie, CHU de Nice, Université Côte d’Azur, Nice, France; Thibaud Chapelle: Département de réanimation médico-chirugicale et anesthésie, CHU de Nice, Université Côte d’Azur, Nice, France.

## Author Contributions

HO, SB-S, and JD designed the study and drafted and revised the manuscript. HO, LM, JC, JF, VB, RL, and ED collected clinical and biological data. HO, LM, and SB-S analyzed and interpreted the data. HO, JC, LM, JF, VB, RL, VE, BS-P, ED, JD, and SB-S provided medical oversight. All authors approved the final version of the manuscript.

## Conflict of Interest

The authors declare that the research was conducted in the absence of any commercial or financial relationships that could be construed as a potential conflict of interest.
