# Supplementary material for: Proteinuria as a Biomarker for COVID-19 Severity
Source: Front Physiol. 2021 Mar 9;12:611772. doi: 10.3389/fphys.2021.611772 (PMC7985082; doi:10.3389/fphys.2021.611772)
Supplement: Supplementary file 1 [file Table_1.pdf]

SUPPLEMENTAL MATERIAL TABLE OF CONTENTS

Supplemental Table 1. Correlation between proteinuria and variables.

Supplemental Table 2. Characteristics of patients with and without acute kidney injury.

Supplemental Table 1. Correlation between proteinuria and variables.

Variables	Spearman's rho	*p* value
Gender	0.32	0.03*
Age, years	0.43	0.003*
Days from onset	-0.02	0.89
Hypertension	0.13	0.39
ACEI/ARB	-0.16	0.30
Diabete mellitus	0.34	0.02
Systolic blood pressure, mmgh	0.23	0.13
Diastolic blood pressure, mmgh	0.09	0.55
Hemoglobin, g/dl	-0.13	0.38
Leukocytes, G/L	0.13	0.40
Lymphocytes, G/L	-0.25	0.10
Neutrophils, G/L	0.22	0.15
Platelet, G/L	-0.008	0.96
C-reactive protein, mg/l	0.41	0.005*
Procalcitonin, ng/ml	0.23	0.16
Albumin, g/L	-0.19	0.25
D-dimer, ng/ml	0.63	<0.0001*
Lactose deshydrogenase, U/L	0.25	0.10
Fibrinogen, g/l	0.25	0.13
Creatinine, µmol/l	-0.06	0.70
BUN, mmol/l	0.23	0.13
Hematuria	0.24	0.12
Leukocyturia	0.03	0.86
In-hospital death	0.12	0.45
Length of stay, days	0.61	<0.0001*

ACEI: angiotensin-converting-enzyme inhibitor; ARB: angiotensin II receptor blockers; BUN: blood urea nitrogen

Supplemental Table 2. Characteristics of patients with and without acute kidney injury.

Variables	AKI (n=12)	Non-AKI (n=33)	*p* value
Age, years	67 (63-72)	59 (47-71)	0.12
Male patients, n (%)	12 (100)	19 (57.6)	0.0006*
Day from illness onset to admission, days	9 (5-13)	8 (5-11)	0.92
Systolic blood pressure, mmgh	128 (120-140)	120 (120-133)	0.37
Diastolic blood pressure, mmgh	76 (70-80)	75 (63-80)	0.73
Respiratory disease, n (%)	3 (25.0)	6 (18.2)	0.61
Hypertension, n (%)	8 (66.7)	9 (27.3)	0.02*
Diabetes mellitus, n (%)	4 (33.3)	8 (24.2)	0.54
Cardiac disease, n (%)	2 (16.7)	3 (9.1)	0.47
Vascular disease, n (%)	2 (16.7)	1 (3.0)	0.10
Active and weaned smoking, n (%)	6 (50)	7 (21.2)	0.06
ACEI, n (%)	0	1 (3.0)	0.54
ARB, n (%)	4 (33.3)	4 (12.1)	0.1
BMI (kg/m²)	28 (26-34)	28 (24-32)	0.55
C Reactive protein, mg/l	102 (86-202)	86 (48-148)	0.16
Procaciltonin, ng/ml	0.44 (0.12-0.79)	0.20 (0.08-0.36)	0.08
D-Dimer, ng/ml	4628 (1501-6110)	1572 (555-4607)	0.07
Proteinuria, g/g	0.66±0.47	0.45±0.47	0.1
Hematuria, n (%)	8 (66.7)	13 (0.39)	0.10
Leukocyturia, n (%)	3 (25.0)	18 (54.5)	0.08
ICU admission, n (%)	8 (66.7)	14 (0.42)	0.15
ARDS, n (%)	6 (50.0)	9 (27.3)	0.15
Length of stay, days	16 (9-25)	10 (5-24)	0.22
Secondary infection, n (%)	6 (50.0)	9 (27.3)	0.15
In-hospital death, n (%)	1 (8.3)	1 (3.0)	0.45

ACEI: angiotensin-converting-enzyme inhibitor; ARB: angiotensin II receptor blockers; ARDS: acute respiratory distress syndrome, BMI: body mass index, BUN: blood urea nitrogen, ICU: intensive care unit.
